# Patient Experience and Predictors of Improvement in a Group Behavioral and Educational Intervention for Individuals With Diabetes and Serious Mental Illness: Mixed Methods Case Study

**DOI:** 10.2196/21934

**Published:** 2021-02-12

**Authors:** Kristina Schnitzer, Corrine Cather, Vanya Zvonar, Alyson Dechert, Rachel Plummer, Kelsey Lowman, Gladys Pachas, Kevin Potter, Anne Eden Evins

**Affiliations:** 1 Department of Psychiatry Massachusetts General Hospital Boston, MA United States; 2 Center for Addiction Medicine Massachusetts General Hospital Boston, MA United States; 3 Center of Excellence for Psychosocial and Systemic Research Department of Psychiatry Massachusetts General Hospital Boston, MA United States; 4 Department of Biostatistics Massachusetts General Hospital Boston, MA United States

**Keywords:** mental disorders, severe, diabetes mellitus, delivery of health care, integrated, behavior and behavior mechanisms, patient education as topic

## Abstract

**Background:**

In a previous study, participation in a 16-week reverse integrated care and group behavioral and educational intervention for individuals with diabetes and serious mental illness was associated with improved glycemic control (hemoglobin A_1c_) and BMI. To inform future implementation efforts, more information about the effective components of the intervention is needed.

**Objective:**

The goal of this study is to identify the aspects of the intervention participants reported to be helpful and to evaluate the predictors of outcomes.

**Methods:**

This study involved qualitative evaluation and post hoc quantitative analysis of a previous intervention. Qualitative data were collected using semistructured interviews with 69% (24/35) of the individuals who attended 1 or more group sessions and 35% (9/26) of the individuals who consented but attended no sessions. Quantitative mixed effects modeling was performed to test whether improved diabetes knowledge, diet, and exercise or higher group attendance predicted improved hemoglobin A_1c_ and BMI. These interview and modeling outcomes were combined using a mixed methods case study framework and integrated thematically.

**Results:**

In qualitative interviews, participants identified the application of health-related knowledge gained to real-world situations, accountability for goals, positive reinforcement and group support, and increased confidence in prioritizing health goals as factors contributing to the success of the behavioral intervention. Improved knowledge of diabetes was associated with reduced BMI (β=–1.27, SD 0.40; *P*=.003). No quantitative variables examined were significantly associated with improved hemoglobin A_1c_ levels.

**Conclusions:**

In this mixed methods analysis of predictors of success in a behavioral diabetes management program, group participants highlighted the value of positive reinforcement and group support, accountability for goals set, and real-world application of health-related knowledge gained. Improved diabetes knowledge was associated with weight loss.

## Introduction

### Background

The lifetime rate of diabetes among people with schizophrenia is at least twice than that seen in the general population [1,2]. A study of Medicaid patients with serious mental illness found an 11.8% prevalence of diabetes [3]. Suboptimal diabetes management in people with serious mental illness is common [4-8] and estimated to cost approximately US $8 billion in the United States annually [8]. High rates of tobacco dependence and poor understanding of diabetes self-management, including diet and physical activity goals, are modifiable factors contributing to the morbidity and mortality associated with diabetes in people with serious mental illness [9-11]. Serious mental illness is used to refer to schizophrenia-spectrum illness, bipolar disorder, or severe major depressive disorder.

Despite high diabetes prevalence and high rates of associated morbidity and mortality, it has been reported that fewer than one-third of adults with serious mental illness are screened for diabetes [8,12]. Moreover, among those diagnosed with diabetes, individuals with mental illness receive less frequent monitoring of glycemic control and cardiovascular risk factors such as hypercholesterolemia than those in the general population, despite recommendations of more frequent monitoring for individuals taking antipsychotic medications [2]. Although providers may assume that people with serious mental illness will be poorly adherent to treatment, adults with serious mental illness have demonstrated good adherence to glucose-lowering medications, disease self-management, and weight loss programs when these treatments have been made available to them [13-16]. At least one large study reported superior adherence to antihyperglycemic medications among people with schizophrenia compared with people without schizophrenia [17].

### Objectives

Our open trial of a group intervention for individuals with serious mental illness and diabetes demonstrated significant improvement in hemoglobin A_1c_ (HbA_1c_) and BMI for participants with serious mental illness and diabetes [18]. Although previous studies of diabetes self-management interventions in this population have reported improvements in BMI, diabetes knowledge, and psychiatric symptoms, to date, no published randomized controlled trials have demonstrated improvements in HbA_1c_ [19,20]. Before designing a larger, more methodologically rigorous controlled trial, we sought to identify unique features of our intervention, successful strategies, key messages retained, and potential predictors of positive outcomes. The purpose of this study is to expand our understanding of successful intervention components through an assessment of patient experience, motivations, and perception of key aspects of the intervention, in addition to quantitative predictors that emerged through repeated surveys during the intervention. As 43% (26/61) of the individuals who consented to this study did not attend any groups, we additionally sought to ascertain barriers to the participation of these individuals to better engage and encourage retention in future iterations of this intervention.

## Methods

### Parent Study Design

The authors designed and tested in an open trial, a behavioral and educational group intervention, modeled on the Diabetes Prevention Program [21], for individuals with comorbid diabetes and serious mental illness that sought to help participants to reduce their HbA_1c_ and BMI by providing health education and support for implementing practical strategies to address the social, economic, and behavioral determinants of health faced by the participants. In the parent study, 61 participants consented to participate and 35 participants attended at least one session of the 16-week intervention over a 2-year period. Details of this trial are given in the study by Schnitzer et al [18].

In this intervention, the modules covered basic diabetes education, diet, exercise, stress reduction, and positive psychology, taught in a simple format with frequent repetition and frequent use of concrete, real-world examples that participants described in the groups. The intervention actively addressed barriers identified by participants to important health-related behaviors and choices. These included barriers to purchasing and preparing healthy food, such as lack of safe or private food storage options, tendency to obtain food from convenience stores and fast-food restaurants, and barriers to exercise (such as discomfort exercising outside because of paranoia or unsafe neighborhoods). Problem solving using the Specific, Measurable, Achievable, Realistic, Timely Framework for individualized behavior goals [22] was tailored to patients’ community environment, for example, how to make healthier choices at the nearby convenience stores and fast-food restaurants. Barriers were addressed in concrete ways with the support of the group to decrease the initial activation demands associated with trying a new behavior on their own. For example, nonsugar-containing beverages were sampled in the group meeting, trips to the Department of Mental Health gym were taken in a group with coleaders exercising alongside the participants, labels were read together in food stores to model how this information might be used to guide the purchase of healthier food options, and a field trip was taken to the nearby affordable hospital cafeteria to demonstrate and practice the identification and purchase of a healthy lunch from available options.

### Current Study—Secondary Analysis Using a Convergent Mixed Methods Design

**Figure 1:**
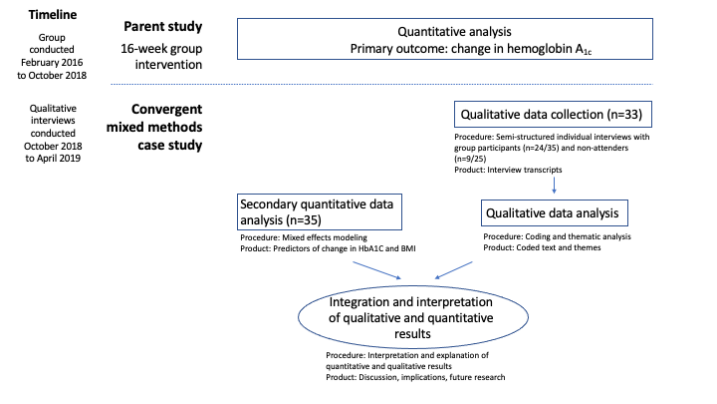
Procedural diagram.

Following the completion of the parent study, the authors conducted semistructured interviews with participants, with the goal of identifying aspects of the intervention that the participants found most helpful. Using an interactive convergent mixed methods study design [23,24], the authors mapped themes that emerged in the qualitative data to quantitative data domains and evaluated these as predictors of improved HbA_1c_ and BMI. The authors also interviewed participants who consented but did not participate to identify barriers to participation and inform implementation of similar interventions in future work (Figure 1). All study procedures were approved by the institutional review board (IRB) of the Massachusetts General Hospital. The parent study IRB was amended to allow the recontacting of previous study participants.

### Qualitative Methods

#### Participants

After completion of the parent study, the authors requested a follow-up interview with all individuals who consented to participate in the study (n=61) about their study experience or barriers to participation. Criteria for eligibility for the initial study included the diagnosis of serious mental illness and HbA_1c_ ≥6.5, HbA_1c_ ≥6 and metformin, or the known diagnosis of diabetes. A total of 33 participants (24 who attended at least one group and 9 who attended no groups) agreed to be interviewed, and the remaining 28 declined to participate or could not be contacted.

#### Exit Interviews

Semistructured individual interviews were designed with open-ended questions querying participants’ overall experience with the behavioral and educational intervention, with questions tailored to reflect their experience as either participating or declining to participate in the group (Multimedia Appendix 1). Interviews were conducted by 3 individuals trained in qualitative interviewing techniques who were not involved in the original study (VZ, RP, and KL). Questions were posed in a neutral and open-ended manner to minimize bias. The interview guide was piloted with 5 participants to assess the clarity of the questions; no subsequent modifications were made. Interviews were conducted in English between October 2018 and April 2019 at the community mental health clinic where the intervention took place and were audio recorded. Individuals received US $10 for their participation.

### Qualitative Analysis

Interviews were transcribed using TranscribeMe Inc to secure transcription services. Analyses were conducted using a grounded theory approach, with the goal of identifying patterns and arranging these in relationships [25,26]. During open coding, a team of 6 researchers met and read 3 transcripts to gain an awareness of the initial thematic content that arose from the interviews. This was followed by analytical coding with grouping of content in consideration of broader meaning and overarching themes. Two codebooks, one for individuals who completed the group and one for individuals who consented to participate but attended no groups, were developed to reflect patterns of response for each of the 2 study paths. These 2 codebooks were revised over the course of 3 meetings.

The finalized codebooks were used in conjunction with NVivo qualitative software version 12.0 (QSR International Pty Ltd) for data organization Qualitative coding was completed by 2 individuals (KS, VZ) with training in qualitative data analysis. KS and VZ coded all transcripts and held weekly recalibration meetings to ensure reliability of coding, reduce coding drift, and resolve discrepancies in coding. Following the completion of coding, the final stage of analysis involved the query of coding reports and a further round of data immersion first individually and then discussed in team meetings to identify content patterns and themes to allow for final interpretation of data. Selected participant quotations were chosen to illustrate the prominent themes expressed by the study sample.

### Quantitative Analysis

The primary outcome for the parent study was change in HbA_1c_ at week 16 [18]. For this secondary analysis, a linear regression with a random intercept for subjects was used to identify potential predictors of 2 physiologic outcomes that were significantly improved from pretreatment to posttreatment, HbA_1c_, and BMI. The authors ran a multivariate analysis within a penalized regression framework, which shrinks estimates slightly toward zero to minimize the risk of false positives and manage any dependencies between predictors. The variables examined included the number of sessions attended, improvement in diabetes knowledge assessed through the Short Diabetes Knowledge Instrument (SDKI) [27], and improvement in diabetes self-care assessed through the Summary of Diabetes Self-Care Activities (SDSCAs) subscales [28]. The SDKI is a 13-item questionnaire developed and validated in a multiethnic sample of individuals >60 years of age to assess an individual’s understanding of diabetes illness and management. The SDSCA measures an individual’s attendance to various aspects of diabetes self-management (general diet, specific diet, and exercise) through assessment of days per week a particular activity is performed.

### Integration of Results and Development of Joint Display

Following qualitative and quantitative data analyses, data were integrated to identify congruencies and discrepancies, allow for meta-inferences, and facilitate richness of data [29-32]. The results were illustrated with a joint display of quantitative predictors mapped to relevant qualitative themes and the resulting mixed method research inference.

## Results

### Sample Characteristics

Full demographic characteristics of the parent study participants are given in the study by Schnitzer et al [18]. Among the sample of individuals who attended at least one group (n=35), the mean age was 53 years, 77% (27/35) were male, 46% (16/35) were white, 34% (12/35) were black, and 20% (7/35) were from *other* race. Among individuals who did not attend any group (n=25), the mean age was 57 years, 60% (15/25) were male, 64% (16/25) were white, 20% (5/25) were black, and 12% (3/25) were from *other* race. Among those who attended at least one group and agreed to participate in the qualitative interview (n=24), the mean age was 52 years, 79% (19/24) were male, 63% (15/24) were white, 33% (8/24) were black, and 4% (1/24) were from *other* race. Among those who did not attend any groups and agreed to participate in the qualitative interview (n=9), the mean age was 50 years, 78% (7/9) were male, 67% (6/9) white, 22% (2/9) black, and 11% (1/9) were from *other* race. Interview participants did not differ in age or racial composition from the parent sample.

### Qualitative Results

The mean duration of interviews was 13 min (SD 6) for those who participated in the groups and 4 min (SD 1) for those who did not participate. Interviews of group participants and nonparticipants are reported separately. Quotes provided include minor editing for flow and clarity.

#### Group Participants

Among those who attended at least one group, 5 major themes developed around the key aspects of the intervention: (1) health-related knowledge gains and application to real-world situations, (2) positive reinforcement from the group, (3) accountability for setting and achieving goals, (4) group support, and (5) increased confidence in the ability to prioritize health goals.

##### Health-Related Knowledge Gains and Application to Real-World Situations

Participants reported learning new information from the group that helped them improve their diabetes self-management. They noted increased confidence in their ability to make changes and said that learning more about how to manage diabetes helped them feel more in control of their health-related decisions. In addition to being able to implement new, healthy behaviors, they also reported being better able to reduce behaviors they learned were unhealthy:

I've learned that many of the foods that I like and have considered relatively innocuous are, in the quantities that I eat them, not conducive to maintaining a good A_1c_. The starches, the breads that I grew up with as someone in an Italian home, pasta and ravioli.

M, 48yo

If I'm going to eat cake, chocolate cake, my sugar level's going to go up, who knows up to how much. I may get dizzy, but if I eat the right things, the ones the group taught me, I can't go wrong.

M, 54yo

Actually, what I did start drinking after I started going to the group was sparkling water or seltzer water. Yeah. I like that. I always get the lime or lemon flavor.

gender fluid, 48yo

And when the clubhouse has their functions, they have all kinds of sweet foods and stuff. You know those little fruit cups? Well, if I go over there for lunch and they're having a fruit cup, I'll say to one of the staff, “I can't have this because it has sugar in it. So, can I have a piece of fruit?”

F, 53yo

When you buy the food, you have to think of how much you need per serving. One time I went in and bought a slab of meat. It was nine servings. They say nine servings on the package. And I had nine servings of meat... I broke it up into nine servings.

F, 70yo

##### Positive Reinforcement From the Group

The importance of positive reinforcement, which the authors define as encouragement from both group leaders and peers, was consistently mentioned by participants as a factor that encouraged their attendance and helped them develop momentum by continuing to build on positive changes:

You build up morale, and you're encouraged to acknowledge people's success. If somebody had, let's say, four days over 10,000 steps, everyone basically applauded. And this is positive reinforcement... I can't stress enough the importance of the positive reinforcement. It almost never felt like a chore to come to the group. I mean, there were days when, owing to my physical limitations by the apnea and the weather, where it was like, “Okay. I got to go in.” But, often enough, that was rewarded with some good thing.

M, 48yo

They were also good in that no one threw the book at you. If you said, “well, I went to the bar and I drank six liters of beer and I had 19 plates of pasta,” they would say, “but one of your days your step count was above 8000.” They would do that... They would say, “More of that and less of the other stuff.” ... They would say, “Here is a good sign.” They'd point out what was going right, and you would get it without having to be beaten over the head with it.

M, 48yo

I got an award for it. Yeah, they gave me a little diabetic award for finishing the course.

M, 42yo

##### Accountability for Setting and Achieving Goals

Many participants commented on the importance of being held accountable to previously set goals, something they did not feel had been a part of efforts to help manage their diabetes before participating in the groups:

You realize, what'd you do well this week? What didn't you do well? We talked about that at the end of the group, and that really helps. That really helps.

M, 68yo

The people, they helped us out a lot. They weighed us. Made sure we were able to keep our weight under control, because they said that was important.

M, 62yo

The very existence of the group certainly helps. The weekly, or every other weekly, depending on when I get in here, accountability to [group leaders] and the other members of the group.

M, 48yo

##### Group Support

Participants reported that being a part of a group entity comprising peers with shared experience caused them to feel less alone and isolated in their efforts to manage their diabetes and improve their overall health:

I mean, here I love people, how they're doing, how their struggles are, and how they're combating it, and how they're dealing with it. It reinforces you, and it's like we're all human. We all fall.

M, 68yo

People like that are family, because you know everybody's in the same category. And it was a nice feeling, you know, you were working at keeping your lifestyle better, living longer, staying healthy.

M, 62yo

Everyone does bring something to the group who shows up and talks candidly. And say, “Oh, yeah. You've discovered that too,” or, “I've done that, too,” or, “Oh, this was a hard week, weather-wise.”

M, 48yo

##### Increased Confidence in the Ability to Prioritize Health Goals

Individuals found reward in and support for making their health a priority and described feeling empowered to continue to make positive changes:

Especially on the food tip. Oh, man. I don't eat as heavy as I used to. I miss it, but my health is more important.

M, 52yo

Yeah. I mean, it just makes you feel like you've got a chance (against diabetes); if you can't beat it, you can control it to where it's not that much of a problem.

M, 68yo

With respect to motivation for group attendance, 58% (14/24) of group participants stated that receiving US $3 was an incentive for group attendance and 63% (15/24) of group participants reported that receiving a free, healthy meal was an incentive for attendance.

#### Group Nonparticipants

Analysis of responses from those who did not attend any groups did not lend itself to a grounded theory approach because of limited and categorical replies. The authors examined categories of responses and summarized the impressions as follows.

Among the 9 individuals interviewed who consented to participate but attended no groups, only 1 reported declining participation because of feeling uncomfortable in a group setting. Two declined because of transportation issues, although both stated that they would have attended if transportation had been provided. Three individuals cited distance as a barrier—of these, 1 stated that he or she would have come if he or she had known about the available US $3 remuneration and 1 stated he or she would have come if remuneration had been US $5. Four individuals cited a time conflict as a barrier, and all the 4 stated that they would have attended if the group intervention were held at a different time.

### Quantitative Results

In regression models, improvement in the diabetes knowledge questionnaire was the only significant predictor of improvement in BMI (β=–1.27, SD 0.40; *P*=.003). There were no identified predictors of improvement in HbA_1c_ (Table 1).

**Table 1:** Predictors of improvement in hemoglobin A_1c_ and BMI.

Variable		Estimate (β)	SD	*P* value
**HbA_1c_^a^**				
	Sessions attended	0.18	0.15	.23
	SDKI^b^	–0.17	0.16	.28
	General diet^c^	0.02	0.16	.89
	Specific diet^c^	0.14	0.15	.34
	Exercise^c^	–0.12	0.16	.43
	BMI	0.26	0.17	.13
**BMI**				
	Sessions attended	0.43	0.38	.25
	SDKI^b^	–1.27	0.40	.003
	General diet^c^	–0.75	0.40	.06
	Specific diet^c^	–0.63	0.39	.11
	Exercise^c^	–0.03	0.40	.93
	HbA_1c_	0.76	0.49	.11

^a^HbA_1c_: hemoglobin A_1c_.

^b^SDKI: Short Diabetes Knowledge Instrument; score range 0-13, with higher scores indicating greater knowledge.

^c^Subscale of summary of diabetes self-care activities measure: multidimensional assessment of diabetes self-management, number corresponding to days per week activity is performed, range 0-7.

### Data Integration

Improvements in diabetes knowledge emerged as the only predictor of outcome supported by both qualitative and quantitative data, insofar as participants self-reported improvement in diabetes knowledge as important on interview and SDKI score predicted improvement in BMI over the course of the 16-week intervention (Table 2). Although participants identified accountability, group support, positive reinforcement, and self-management (health prioritization and improved self-confidence) as helpful, neither session attendance rate nor score improvements on a measure of diet and exercise self-care predicted improvement in HbA_1c_ or BMI.

**Table 2:** Joint display of quantitative predictors mapped to relevant qualitative themes and resulting mixed methods research inference for group attenders.

Quantitative measures	Predictive of Improvement in A_1c_ or BMI	Emergent qualitative themes	MMR^a^ inference
Improvements in diabetes knowledge	Yes	Health-related knowledge gains and application to real-world situations	Convergence
Session attendance	No	Accountability in goal setting Group support Positive reinforcement	Divergence Importance of factor expanded through qualitative data
Diet and exercise self-care	No	Health prioritization and improved self-confidence	Divergence Importance of factor expanded through qualitative data

^a^MMR: Mixed Methods Research.

## Discussion

### Principal Findings

Although the open trial demonstrated significant improvement in HbA_1c_ with a diabetes self-management intervention, none of the variables the authors investigated (attendance, change in diabetes knowledge, and change in diabetes self-care) emerged as a significant predictor of this improvement. Of the predictors investigated, only improvement in diabetes knowledge predicted improved BMI. Qualitative interviews shed light on additional thematic and structural group components that participants viewed as key to their group engagement and success.

Knowledge gains related to diabetes, as measured by SDKI, and knowledge gains related to health-promoting behaviors identified through qualitative interviews appear to be an important piece of facilitating behavior change commensurate with improved diabetes self-management. SDKI improvements did not, however, significantly predict changes in HbA_1c_, the primary outcome of the study. This suggests that although objective knowledge gains may be important, these are not the only factors driving improved diabetes self-management, and others may be learned through capture of emergent qualitative themes.

Although session attendance was not predictive of outcomes, participants identified accountability, group support, and positive reinforcement attained through group attendance as crucial to their success. This suggests that the absolute number of sessions attended may be less important than a patient’s ability to engage meaningfully with the intervention, such as the individual’s capacity to give and receive positive reinforcement and interact in a group setting. Our results align with work supporting the value of peer support in promoting self-management of mental health [33-35], which underscore the importance of peer support, encouragement, hope, and empowerment. Novel interventions show promise in the management of physical health for individuals with serious mental illness, which incorporate peer supporters as change agents [36], and one future direction is to consider employing peers to help deliver diabetes self-management interventions.

Of note, measures of psychiatric symptom severity were not collected during this study but could be important proxies for an individual’s ability to benefit from such an intervention, with negative symptoms and cognitive limitations likely presenting barriers to meaningful engagement. Similarly, although scores on diet and exercise self-care scales were not significantly predictive of group performance, improved self-confidence and empowerment to prioritize health were frequently coded themes in the interviews. This has important implications for how the authors may operationalize success from such interventions, including the potential importance of measuring additional outcomes and process variables such as hope, self-efficacy, and empowerment [37].

Payment for group attendance was noted as a moderate incentive for individual attendance for approximately half of the interviewed participants who reported that they used it primarily for transportation or for purchase of food. Similarly, receiving lunch as part of the group appeared to be a moderate incentive for over half of the interviewed participants. Although these 2 contingencies were helpful with retention, most participants cited aspects of the group itself (camaraderie and support) and the positive health behavior changes they were making as their primary incentive for continued participation.

The most cited reason for not attending any group was time conflict, followed by distance and transportation difficulties. Importantly, only one individual stated that he or she declined to participate because of the group format itself, suggesting that, if such programs are made more available, this population is largely willing to participate in a group setting.

This intervention was delivered in a community mental health center where the majority of patients received treatment. Reverse integrated care interventions, defined as medical care delivered in behavioral health care settings, have the potential for high impact on medical management in this population, as individuals with serious mental illness visit psychiatric providers more frequently than their primary care providers and often feel more comfortable in their behavioral health care settings [38]. Moreover, familiarity and knowledge of strengths and self-efficacy of individuals with serious mental illness whom they treat may enable psychiatric care providers to be particularly effective in supporting health behavior change [39,40]. In addition, increasing implementation of behavioral health homes and electronic medical records has increased the ease of communication between psychiatric and medical providers, which may enhance the feasibility and impact of reverse integrated care models for improving management of chronic medical illnesses in people with serious mental illness.

Future studies of diabetes and health management in this population would do well in considering the increasing role and, at times, the necessity of incorporating virtual care models into interventions such as the group intervention presented here, when traditional in-person models may not be possible. Studies have demonstrated the potential role of text messaging and mobile apps in increasing engagement and symptom tracking for individuals with psychosis [41,42] and a growing role for virtual components for enhancing diabetes self-management and support [43,44]; however, to our knowledge, no studies have examined a virtual group intervention for diabetes or a mobile intervention for diabetes tailored for individuals with serious mental illness.

### Limitations

To minimize potential threats to validity, the same sample was used for quantitative and qualitative analyses, a joint display was developed to depict congruency and discrepancy, and both quantitative and qualitative results were reported. The authors maximized variation by approaching all individuals who completed the study for interviews, in addition to individuals who did not attend any groups. As our sample contained individuals who were stable but with serious psychiatric illness and multiple medical comorbidities, this study may be applicable to populations with heavy medical and psychiatric burden served in the broader community, although we noted that in this study, only a small sample of individuals, at 1 community mental health center in Boston, were sampled. Several interviews were notably very brief, particularly among those who did not attend groups, although they were included as they provided information on rationale for group nonattendance and hold the potential to inform future implementation efforts. Researcher bias is possible in this open-label study, as one of the study clinicians who delivered the intervention also participated in coding the qualitative data. Bias was reduced by having a researcher not involved in the intervention conduct the qualitative interviews.

### Conclusions

This study highlighted the group model using a combined educational and behavioral approach as a potentially valuable mechanism for health-related behavior change among individuals with serious mental illness who are affected by disproportionate morbidity and premature mortality from illnesses with large modifiable health behavior contributors. Participants highlighted the value of the group model and positive reinforcement, accountability, and real-world application of knowledge gained for improving health-related knowledge, behavior, and outcomes. This intervention, in which both psychiatric and medical professionals were on a team with group members to guide and support, provides an example of participatory medicine in practice in the group setting. Larger scale reverse integrated care controlled trials for individuals with diabetes and serious mental illnesses are needed and would do well to incorporate the aspects of positive reinforcement, patient accountability for individual goals set in the program, and real-world application of the educational concepts highlighted here.

## Appendix

Multimedia Appendix 1 Semistructured interview guide.

## Abbreviations

HbA_1c_: hemoglobin A_1c_
IRB: Institutional Review Board
SDKI: Short Diabetes Knowledge Instrument
SDSCA: Summary of Diabetes Self-Care Activity

## References

[1] De Hert M, Mauri M, Shaw K, Wetterling T, Doble A, Giudicelli A, Falissard B (2010). The METEOR study of diabetes and other metabolic disorders in patients with schizophrenia treated with antipsychotic drugs. I. Methodology. Int J Methods Psychiatr Res.

[2] DE Hert M, Schreurs V, Vancampfort D, VAN Winkel R (2009). Metabolic syndrome in people with schizophrenia: a review. World Psychiatry.

[3] Banta JE, Morrato EH, Lee SW, Haviland MG (2009). Retrospective analysis of diabetes care in California Medicaid patients with mental illness. J Gen Intern Med.

[4] Frayne SM, Halanych JH, Miller DR, Wang F, Lin H, Pogach L, Sharkansky EJ, Keane TM, Skinner KM, Rosen CS, Berlowitz DR (2005). Disparities in diabetes care: impact of mental illness. Arch Intern Med.

[5] McGinty EE, Baller J, Azrin ST, Juliano-Bult D, Daumit GL (2015). Quality of medical care for persons with serious mental illness: a comprehensive review. Schizophr Res.

[6] Kreyenbuhl J, Dickerson FB, Medoff DR, Brown CH, Goldberg RW, Fang L, Wohlheiter K, Mittal LP, Dixon LB (2006). Extent and management of cardiovascular risk factors in patients with type 2 diabetes and serious mental illness. J Nerv Ment Dis.

[7] Mangurian CV, Schillinger D, Newcomer JW, Vittinghoff E, Essock SM, Zhu Z, Dyer WT, Schmittdiel JA (2018). Diabetes and prediabetes prevalence by race and ethnicity among people with severe mental illness. Diabetes Care.

[8] Mangurian C, Newcomer JW, Modlin C, Schillinger D (2016). Diabetes and cardiovascular care among people with severe mental illness: a literature review. J Gen Intern Med.

[9] Hartz SM, Pato CN, Medeiros H, Cavazos-Rehg P, Sobell JL, Knowles JA, Bierut LJ, Pato MT, Genomic Psychiatry Cohort Consortium (2014). Comorbidity of severe psychotic disorders with measures of substance use. JAMA Psychiatry.

[10] Chwastiak LA, Freudenreich O, Tek C, McKibbin C, Han J, McCarron R, Wisse B (2015). Clinical management of comorbid diabetes and psychotic disorders. Lancet Psychiatry.

[11] Teasdale SB, Ward PB, Samaras K, Firth J, Stubbs B, Tripodi E, Burrows TL (2019). Dietary intake of people with severe mental illness: systematic review and meta-analysis. Br J Psychiatry.

[12] Mangurian C, Newcomer JW, Vittinghoff E, Creasman JM, Knapp P, Fuentes-Afflick E, Schillinger D (2015). Diabetes screening among underserved adults with severe mental illness who take antipsychotic medications. JAMA Intern Med.

[13] Piette JD, Heisler M, Ganoczy D, McCarthy JF, Valenstein M (2007). Differential medication adherence among patients with schizophrenia and comorbid diabetes and hypertension. Psychiatr Serv.

[14] Krein SL, Bingham CR, McCarthy JF, Mitchinson A, Payes J, Valenstein M (2006). Diabetes treatment among VA patients with comorbid serious mental illness. Psychiatr Serv.

[15] Daumit GL, Dalcin AT, Jerome GJ, Young DR, Charleston J, Crum RM, Anthony C, Hayes JH, McCarron PB, Khaykin E, Appel LJ (2011). A behavioral weight-loss intervention for persons with serious mental illness in psychiatric rehabilitation centers. Int J Obes (Lond).

[16] Daumit GL, Dickerson FB, Wang N, Dalcin A, Jerome GJ, Anderson CAM, Young DR, Frick KD, Yu A, Gennusa JV, Oefinger M, Crum RM, Charleston J, Casagrande SS, Guallar E, Goldberg RW, Campbell LM, Appel LJ (2013). A behavioral weight-loss intervention in persons with serious mental illness. N Engl J Med.

[17] Kreyenbuhl J, Dixon LB, McCarthy JF, Soliman S, Ignacio RV, Valenstein M (2010). Does adherence to medications for type 2 diabetes differ between individuals with vs without schizophrenia?. Schizophr Bull.

[18] Schnitzer K, Cather C, Thorndike AN, Potter K, Freudenreich O, MacLaurin S, Vilme M, Dechert A, Wexler D, Evins AE (2020). Improved glycemic control in adults with serious mental illness and diabetes with a behavioral and educational intervention. Psychiatr Serv.

[19] McKibbin CL, Golshan S, Griver K, Kitchen K, Wykes TL (2010). A healthy lifestyle intervention for middle-aged and older schizophrenia patients with diabetes mellitus: a 6-month follow-up analysis. Schizophr Res.

[20] Sajatovic M, Gunzler DD, Kanuch SW, Cassidy KA, Tatsuoka C, McCormick R, Blixen CE, Perzynski AT, Einstadter D, Thomas CL, Lawless ME, Martin S, Falck-Ytter C, Seeholzer EL, McKibben CL, Bauer MS, Dawson NV (2017). A 60-week prospective rct of a self-management intervention for individuals with serious mental illness and diabetes mellitus. Psychiatr Serv.

[21] Diabetes Prevention Program (DPP) Research Group (2002). The Diabetes Prevention Program (DPP): description of lifestyle intervention. Diabetes Care.

[22] Bovend'Eerdt TJH, Botell RE, Wade DT (2009). Writing SMART rehabilitation goals and achieving goal attainment scaling: a practical guide. Clin Rehabil.

[23] Moseholm E, Fetters MD (2017). Conceptual models to guide integration during analysis in convergent mixed methods studies. Methodological Innovations.

[24] Fetters MD, Curry LA, Creswell JW (2013). Achieving integration in mixed methods designs-principles and practices. Health Serv Res.

[25] Strauss A, Corbin J (1998). Basics of Qualitative Research : Techniques and Procedures for Developing Grounded Theory.

[26] Miles MB, Huberman AM (1984). Qualitative Data Analysis: A Sourcebook of New Methods.

[27] Quandt SA, Ip EH, Kirk JK, Saldana S, Chen S, Nguyen H, Bell RA, Arcury TA (2014). Assessment of a short diabetes knowledge instrument for older and minority adults. Diabetes Educ.

[28] Toobert DJ, Hampson SE, Glasgow RE (2000). The summary of diabetes self-care activities measure: results from 7 studies and a revised scale. Diabetes Care.

[29] Creswel JW, Klassen AC, Clark VLP, Smith KC (2013). Best practices for mixed methods research in the health sciences. NIH Office of Behavioral and Social Sciences. (2018). Best practices for mixed methods research in the health sciences (2nd ed). Bethesda: National Institutes of Health.

[30] Tashakkori A, Teddlie C, Charles B (1998). Mixed methodology: Combining Qualitative and Quantitative Approaches.

[31] Collins KM, Onwuegbuzie AJ, Sutton IL (2006). A model incorporating the rationale and purpose for conducting mixed-methods research in special education and beyond. Learning disabilities: A Contemporary Journal.

[32] Guetterman TC, Fetters MD, Creswell JW (2015). Integrating quantitative and qualitative results in health science mixed methods research through joint displays. Ann Fam Med.

[33] Walker G, Bryant W (2013). Peer support in adult mental health services: a metasynthesis of qualitative findings. Psychiatr Rehabil J.

[34] Repper J, Carter T (2011). A review of the literature on peer support in mental health services. J Ment Health.

[35] Fortuna KL, Naslund JA, Aschbrenner KA, Lohman MC, Storm M, Batsis JA, Bartels SJ (2019). Text message exchanges between older adults with serious mental illness and older certified peer specialists in a smartphone-supported self-management intervention. Psychiatr Rehabil J.

[36] Aschbrenner KA, Naslund JA, Shevenell M, Mueser KT, Bartels SJ (2016). Feasibility of behavioral weight loss treatment enhanced with peer support and mobile health technology for individuals with serious mental illness. Psychiatr Q.

[37] Bartels SJ, DiMilia PR, Fortuna KL, Naslund JA (2018). Integrated care for older adults with serious mental illness and medical comorbidity: evidence-based models and future research directions. Psychiatr Clin North Am.

[38] Daumit GL, Pratt LA, Crum RM, Powe NR, Ford DE (2002). Characteristics of primary care visits for individuals with severe mental illness in a national sample. Gen Hosp Psychiatry.

[39] Smith JD, Mittal D, Chekuri L, Han X, Sullivan G (2017). A comparison of provider attitudes toward serious mental illness across different health care disciplines. Stigma and Health.

[40] Lester H, Gask L (2006). Delivering medical care for patients with serious mental illness or promoting a collaborative model of recovery?. Br J Psychiatry.

[41] D'Arcey J, Collaton J, Kozloff N, Voineskos AN, Kidd SA, Foussias G (2020). The use of text messaging to improve clinical engagement for individuals with psychosis: systematic review. JMIR Ment Health.

[42] Firth J, Torous J (2015). Smartphone apps for schizophrenia: a systematic review. JMIR Mhealth Uhealth.

[43] Baptista S, Wadley G, Bird D, Oldenburg B, Speight J, My Diabetes Coach Research Group (2020). Acceptability of an embodied conversational agent for type 2 diabetes self-management education and support via a smartphone app: mixed methods study. JMIR Mhealth Uhealth.

[44] Baptista S, Wadley G, Bird D, Oldenburg B, Speight J, My Diabetes Coach Research Group (2020). User experiences with a Type 2 diabetes coaching app: qualitative study. JMIR Diabetes.

