# Supplementary figures and images for: Patient Experience and Predictors of Improvement in a Group Behavioral and Educational Intervention for Individuals With Diabetes and Serious Mental Illness: Mixed Methods Case Study
Source: J Particip Med. 2021 Feb 12;13(1):e21934. doi: 10.2196/21934 (PMC7910121; doi:10.2196/21934)

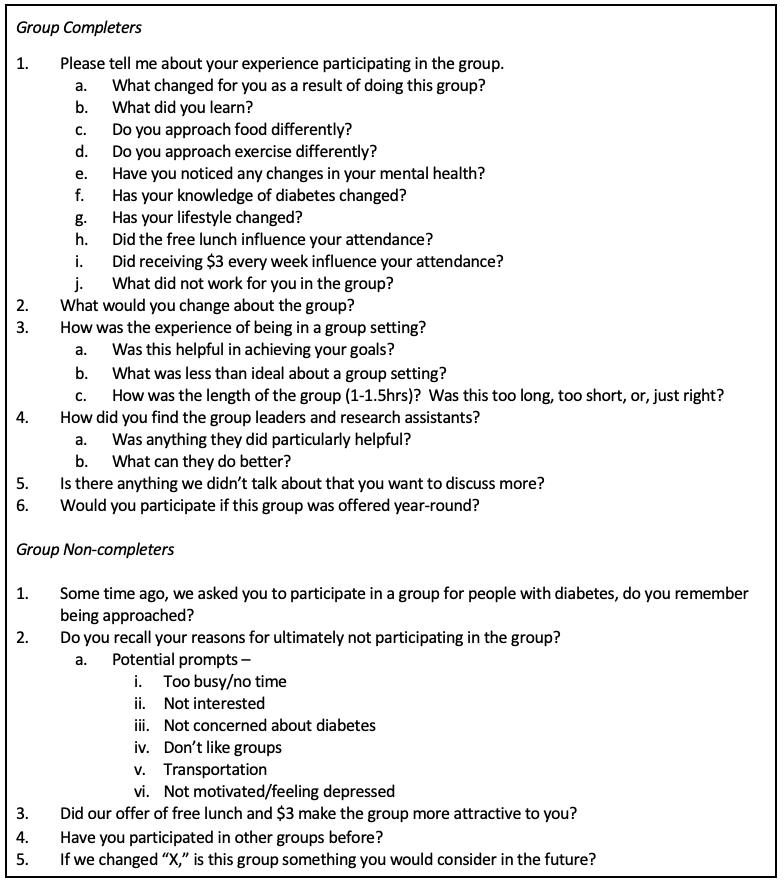

Supplement: Multimedia Appendix 1 [file jopm_v13i1e21934_app1.docx]
